# Phenolic compounds occurrence and human health risk assessment in potable and treated waters in Western Cape, South Africa

**DOI:** 10.3389/ftox.2023.1269601

**Published:** 2024-01-04

**Authors:** Nkosiyenzile Londiwe Mhlongo, Michael Ovbare Akharame, Omoniyi Pereao, Izanne Susan Human, Beatrice Olutoyin Opeolu

**Affiliations:** ^1^ Department of Environmental and Occupational Studies, Cape Peninsula University of Technology, Cape Town, South Africa; ^2^ Department of Environmental Management and Toxicology, University of Benin, Benin-City, Nigeria; ^3^ Environmental Chemistry and Toxicology Research Group, Cape Peninsula University of Technology, Cape Town, South Africa

**Keywords:** potable water, wastewater, 4-chlorophenol, 2,4-dichlorophenol, mutagenicity, risk assessment, human health risk

## Abstract

Phenolic pollutants from industrial and agricultural activities pose a major threat to the world’s potable water supply. The persistent micro-pollutants often find their way into drinking water sources with possible adverse human health implications. In this study, bottled water, tap water, and wastewater treatment plant (WWTP) effluent samples from the Boland region of the Western Cape, South Africa were assessed to determine 4-chlorophenol (4-CP) and 2,4-dichlorophenol (2,4-DCP) levels using HPLC/DAD instrumentation. The selected area is known for its vast agricultural ventures and wineries. Evaluation of the human health risk (cancer risk) for the pollutants was conducted using the hazard quotient (HQ). The Ames mutagenicity test was also conducted using the *Salmonella typhimurium* T98 and T100 strains and the S9 activation enzyme. Trace levels of the phenolics were detected in the samples with a range of 9.32 × 10^−7^—1.15 × 10^−4^ mg/L obtained for 4-CP, and 8.80 × 10^−7^—1.72 × 10^−4^ mg/L recorded for 2,4-DCP. Both compounds had levels below the limit of 0.01 mg/L prescribed by South African legislation. The assessed HQ for the phenolic concentrations indicates a low level of potential ecological risk and none of the samples had a cancer risk value that exceeded the regulatory limit. The possibility of the analyzed samples causing cancer is unlikely, but non-carcinogenic adverse effects were found. Strong mutagenicity was observed for the T98 strains with a potential ability to cause mutation toward the insertion or deletion of a nucleotide. The T100 bacterial strain showed very slight mutagenicity potential, however, it is unlikely to cause any mutation. The levels of phenolics in the potable water samples may pose a significant threat to human health. Hence, screening persistent organic chemicals in potable water sources and evaluating their potential human health effects is pertinent to prevent associated health challenges.

## 1 Introduction

Securing a safe, economical, accessible, and reliable water supply is currently being threatened by the anthropogenic deposition of organic chemicals and micro-pollutants in the different environmental matrices (Akharame et al., 2022). The deposition of these organic compounds in surface waters and underground aquifers poses challenges to the availability of potable water resources. Industries and agricultural ventures are the major culprits responsible for the organic chemicals and micro-pollutants proliferation in the environment. Many industrial facilities and municipalities operate wastewater treatment plants (WWTPs) as a regulatory measure to mitigate the presence of various contaminants in their effluent discharges (Akharame and Ogbebor, 2023). However, the presence of some chemicals and micro-pollutants in the influent streams poses a serious challenge to the treatment capabilities of WWTPs.

The presence of organic chemicals and micro-pollutants is a serious barrier faced by treatment plants, as pollutants reduce the adequacy of treated wastewater to be discharged into water bodies or used as a source of drinking water. Literature reports suggest that the traditional wastewater treatment solutions available in South Africa are largely insufficient to treat wastewater before discharge or reuse (Afolabi et al., 2018; Edokpayi et al., 2018; Olabode et al., 2020; Pereao et al., 2021). Hence, many municipalities and provinces in South Africa have taken the initiative to upgrade some WWTPs to new membrane bioreactor systems which may provide an improved capability to treat the influent (Zhang et al., 2021). Despite the laudable drive to enhance the effluent treatment processes and capabilities in the country, there is the possibility that the persistent organic chemicals can still find their way into drinking water sources which may pose a threat to human health. Phenols and their derivatives are examples of persistent organic chemicals currently regarded as priority pollutants due to their increasing presence in water sources and their potential to cause adverse health effects (Bakthavatsalam, 2019). Ingestion of water with high levels of phenolics can potentially cause unintentional muscle contractions (muscle tremors), walking abnormalities, and death (ATSDR, 2008); more so, they possess endocrine-disrupting characteristics (Alshabib and Onaizi, 2019; Bakthavatsalam, 2019). Levels of phenolics have been reported in environmental media such as the atmosphere, sediments, plants, animals, and human bodies (Lv et al., 2019; Li et al., 2020; Liu and Mabury, 2020; Mykhailenko et al., 2020; Wang et al., 2020; Sun et al., 2022). Particularly, phenolics have been detected in drinking water, rainwater, groundwater, surface waters, urban runoff, as well as industrial effluents (ATSDR, 2008). Phenolic compound deposition in the environmental matrices is aggravated by the production and application of numerous pesticides and the generation of municipal and industrial effluents (Raza et al., 2019; Krithiga et al., 2022; Okoro et al., 2022).

Consequently, this study is focused on two phenolic compounds - 4-chlorophenol (4-CP) and 2,4-dichlorophenol (2,4-DCP). The 4-CP contaminant has been reported to possess carcinogenic and mutagenic properties (Haddadi and Shavandi, 2013; Hidalgo et al., 2013; Lim et al., 2013), and the European Union Directive classifies it as a dangerous substance (Orejuela and Silva, 2002). It is largely present in many agro-chemicals used in commercial farming and is a by-product formed from winery operations which are the main economic activities in the research area. For 2,4-DCP, is listed as part of the eleven phenols deemed as critical contaminants by the United States Environmental Protection Agency (Mohd, 2022). The 2,4-DCP compound is used in copious quantities in the manufacture of certain herbicides and preservatives, such as 2,4-dichlorophenoxyacetic acid and pentachlorophenol (Estevinho et al., 2007; Moszczyński and Białek, 2012; Kuśmierek et al., 2016), and it is also a by-product formed during the chlorination process utilized for water disinfection. The ubiquitous occurrences of chlorinated phenolic compounds in the various environmental matrices elicited the motivation for the investigation. Additionally, our research team has carried out several investigations targeting phenolic compounds due to their endocrine-disrupting potentials. This includes investigating their degradation route during ozonation processes (Oputu et al., 2019; Akharame et al., 2020), and proffering remediation measures using heterogeneous catalytic approaches (Oputu et al., 2015a; Oputu et al., 2015b; Akharame et al., 2022). Phenolic compounds’ environmental contamination pathways include the manufacturing of preservatives, pulp and paper, pesticides and dyes, wines, and other phenol-based compounds (Xu et al., 2017; Raza et al., 2019; Yahaya et al., 2019). The compounds pose threatening human health issues due to their quantum of industrial and agricultural usage which has given rise to accumulations in the environment. The levels of 4-CP and 2,4-DCP were assessed in the bottled water, tap water, and wastewater treatment plant (WWTP) effluent samples from the Boland region of the Western Cape, South Africa. A correlation with the human health hazard quotient was created to assess the overall carcinogenic health effect of the water samples. The Ames mutagenicity test was conducted to determine whether the brands of bottled water, tap water, and WWTP effluent could potentially cause a mutagen. Screening of persistent organic chemicals in potable water sources and evaluating their potential human health effects is pertinent to prevent associated health challenges.

## 2 Materials and methods

### 2.1 Chemical and standards

The phenolic compounds 4-CP (99%), 2,4-DCP (99%), HPLC grade acetonitrile (>98%), Supelco C18-E cartridges (500 mg/12 mL of adsorbent) were obtained from Sigma Aldrich (South Africa). Milli-Q water-18 Ω (Milli-Q Academic, France) was utilized for all the analytical work.

### 2.2 Sampling and extraction procedure of phenols

The study was conducted in Cape Town and the surrounding Boland region of South Africa. The selected area is known for its agricultural processes and wineries. The water samples were collected in the autumn and winter months following standard procedures (APHA, 1998). The bottled water (four brands) was purchased from grocery stores, the tap water samples were collected from a University campus, and the influent and effluent samples were taken from a WWTP (a membrane bioreactor system) located in the region. The water samples were collected using sterile 500 mL amber bottles. The samples were kept in an ice chest for preservation en route to the laboratory. The samples from the WWTP were filtered by using a 0.22 µm polyethersulphone membrane syringe filter and then refrigerated at 4°C.

The extraction procedure for the phenolics was done following the method previously described by (Olujimi et al., 2011). The Sulpeco C18-E cartridges were conditioned with 8.5 mL *n*-hexane: acetone (50:50 v/v), 8.5 mL methanol, and 15 mL Milli-Q water, respectively. Adjustment of the water samples’ pH to 2.5 was done using hydrochloric acid before filtering through the conditioned cartridges. This was followed by channeling 5 mL of Milli-Q water through the cartridges and holding it under a vacuum for 30 min to dry (−70 kPa). The analyte elution from the cartridges was achieved by using 3.5 mL of methanol, and 3.5 mL of n-hexane: acetone (50:50 v/v) in a glass flask, respectively. Thereafter, the drying process with a gentle stream of nitrogen was effected, and aliquots from the solution were analyzed with HPLC instrumentation. The concentrations of the assayed analytes were determined using external calibration standards.

### 2.3 HPLC instrumentation and chromatographic conditions

The separation and identification of the compounds was done using the HPLC instrumentation (Waters Corporations, United States). The instrument setup consists of a terminal solvent delivery system (Waters 1525 binary HPLC pump), an autosampler (Waters 2707 auto-sampler), photodiode array detector (Waters 2487 dual λ absorbance detector), using the Breeze software^™^ as the analytical software. An Ace 5 C18 column (150 × 4.6 mm i. d) was utilized to achieve the separation of the compounds, with the elution done using optimized binary gradients (SI Table 1). Milli-Q water (“A”) and acetonitrile (“B”) were the mobile phases operated at a flow rate of 1 mL/min for the chromatographic separations, while the detection of the compounds was at 280 nm. Pre-conditioning of the chromatographic system was effected by a continuous flow of the solvents for 30 min to obtain a stable baseline signal. Thereafter, the assays were done by the injection of 20 mL of the analytes and standards at an operating temperature of 25°C. The retention time values and the UV-spectral of the target analytes were used for the compound identification.

### 2.4 Human health risk assessment

#### 2.4.1 Cancer risk assessment for 4-CP and 2,4-DCP exposure

The United States Environmental Protection Agency (USEPA) health risk assessment equations used for estimating exposure to phenols were adopted for this study (Rand and Mabury, 2017; Okoro et al., 2022). The cancer risk assessment was evaluated with the average daily dose (ADD) and hazard quotient (HQ) using Eqs. 1–3. The values used for the exposure calculation are presented in Supplementary Table S2.

$$ADD=\left({IR}^{*}C^{*}{EF}^{*}ED\right)/\left({BW}^{*}AT\right)$$ (1)

Where IR = Ingestion Rate, C= Concentration, EF = Exposure Frequency, ED = Exposure Duration, BW = Body Weight, AT = Averaging Time (Life Expectancy)

$$Hazard\;Quotient\;\left(HQ\right)=\left(ADD\right)/\left(RfD\right)$$ (2)

Where RfD is the Reference Dose (IRIS-USEPA)

$$Cancer\;risk={SF}^{*}ADD$$ (3)

where SF is the slope factor.

#### 2.4.2 Mutagenicity testing (Ames test)

The mutagenicity assay (Ames test) was carried out using ‘Muta-ChromoPlate^™^ Bacterial Strain Kit with S9 Activation TM Version 2.1’ for 6 days. The testing followed the 96-well microplates protocol of the *Salmonella typhimurium* test developed for investigating mutagenic constituents in soluble extracts from different environmental matrices (air, water, and land or sediment), food components, chemicals, and cosmetics (Ames et al., 1975). The following reagents in their required measures were utilized: Davis-Mingoli salts 22 mL (A), D-glucose 10 mL (B), bromocresol purple 7 mL (C), D-biotin 4 mL (D), L-histidine 200 mL (WP2 strains substitute L-tryptophan, 100 µL) (E), sterile distilled water, 120 mL (F), growth medium 5 mL (G), and ampicillin 100 mL (V). The composition of the S9 mix is shown in Table 1. In preparing the S9 mix, the constituents S9A to S9E were added in the reverse order before the addition of the supernatant of a liver homogenate (S9F); the addition sequence is to ensure that the S9 fraction is added to a buffered solution (Maron and Ames, 1983). The prepared S9 mix was immediately kept on ice to prevent loss of activity.

**TABLE 1:** Composition of S9 mix.

Constituent	Volume (mL)
S9A: MgCl_2_ + KCl solution	0.96
S9B: Glucose-6-phosphate	0.22
S9C: NADP	1.94
S9D: Phosphate buffer	23.96
S9E: Sterile water	20.32
S9F: S9 fraction (hydrate with 2.1 mL of sterile H_2_O)	0.60
**Total**	**48.00**

##### 2.4.2.1 Lyophilized test strains and standard mutagens

The lyophilized test strains and standard mutagen assays were composed of T98, T100, NaN_3_, 110 mL—for use with TA 100, 2-nitro fluorene (2-NF, 110 μL)—for use with TA 98, and 2-amino anthracene (2-AA, 110 μL)—for use with S9 activation kits.

##### 2.4.2.2 Hydration of dried bacteria and incubation

Prior hydration of the dried bacteria and its incubation was initiated a night before the assay. The procedure commences with transferring 10 mL of reagent ampicillin (V) into the growth media (G) and followed by mixing with the lyophilized bacteria (T98 and T100). Thereafter, the growth media was transferred aseptically into vials containing bacteria and mixed. Incubation of the mixed lyophilized bacteria was done for 18 h at 37°C. Visualization to confirm the bacteria growth was done before proceeding with the assay. The ampicillin included in the mutagenicity assay serves as a marker for the presence of plasmid; the plasmid tends to confer ampicillin resistance which is a convenient marker (Maron and Ames, 1983; Tejs, 2008). The TA98 and TA100 strains were tested on the same plate as a control for ampicillin activity.

##### 2.4.2.3 Aqueous sample dilutions

The samples to be tested were filtered through a 0.22 µm membrane filter for sterilization purposes. Subsequently, the sample mix setup of the Muta-ChromoPlate™ assay with S9 activation for T98 and T100 bacterial strains was prepared in 50 mL sterile tubes as shown in Table 2.

**TABLE 2:** Experimental setup of the Muta-ChromoPlate™ Assay with S9 activation for T98 and T100 bacterial strains.

Treatment plate (1–12)	Standard	Sample	Water	Reaction mix	S9 mix	Bacteria (5 µL)
Blank (sterility check)	-	15.5	0	2.5	2.0	-
Background	-	-	15.5	2.5	2.0	+
Positive control	0.1	-	15.5	2.5	2.0	+
WWTP effluent I (with S9)	-	15.5	0	2.5	2.0	+
WWTP effluent I	-	15.5	0	2.5	0.0	+
WWTP effluent II	-	3.0	12.5	2.5	2.0	+
Tap water I (with S9)	-	15.5	0	2.5	2.0	+
Tap water I	-	15.5	0	2.5	0.0	+
Tap water II	-	3.0	12.5	2.5	2.0	+
**4-CP**						
Bottled water A-I (with S9)	-	15.5	0	2.5	2.0	+
Bottled water A-I	-	15.5	0	2.5	0.0	+
Bottled water A-II	-	15.5	0	2.5	0.0	+
**2,4-DCP**						
Bottled water B-I (with S9)	-	3.0	12.5	2.5	2.0	+
Bottled water B-I	-	15.5	0	2.5	2.0	+
Bottled water B-II	-	3.0	12.5	2.5	2.0	+

Bold values indicate the sectioning of the tables.

##### 2.4.2.4 Preparation of treatments (with and without S9 activation enzyme) and Muta-ChromoPlateTM assay

The reaction mixture (2.5 mL) was transferred aseptically into all the holding vials containing the test samples, followed by the addition of 15.5 mL of the sterile filtered samples or dilutions to be analyzed. The S9-activation enzyme experiments proceeded with the addition of 2.0 mL of S9 mix to each vial requiring S9 activation only. This was followed by the addition of the reaction mixture (2.5 mL) and 15.5 mL of the samples as shown in Table 2. All the assay vials containing materials to be assessed had 5 mL of bacterial test strain broth culture (*S. typhimurium* T98 and TA100) added to ensure that the bacteria were completely suspended. The resulting mixtures in all the vials were poured into a sterile boat and 200 mL of the mixture was dispensed into a 96-well sterile microplate using a multi-channel pipette. Labeling of the plates for facile identification and separation of the bacterial strains was carried out. Incubation in aseptic-sealed plastic bags was done for 3–6 days at 37°C. The addition of the S9-activation enzyme is predicated on the fact that carcinogens are not directly carcinogenic, they become active after metabolism. The S9 mix (consisting of a 9000 supernatant fraction of liver homogenate from rats and other components) strongly induces several xenobiotic metabolizing enzymes (Hengstler and Oesch, 2001). Essentially, the S9 mix is added to the reaction mixture to provide a favorable condition for the activation of the metabolism of the carcinogen.

### 2.5 Interpretation of results

The statistical significance difference was determined for each treatment plate using the method described by Mortelmans and Zeiger (2000). The 96-well microplate method described by Gilbert (1980) was used for scoring mutagenicity.

## 3 Results

### 3.1 Phenol levels in WWTP effluent and potable water samples

The HPLC chromatograms, calibration data, and curves for 4-CP and 2,4-DCP are presented in Supplementary Figures S1, S2A, S2B, and Supplementary Table S3, respectively. The calibration curve *R* ^2^ values for both compounds were >0.999 which indicates the suitability of the method used for the analysis. The retention time for 4-CP and 2,4-DCP were 11.7 min and 14.1 min, respectively. The phenolic levels recorded in the tap water, bottled water, and WWTP effluent are presented in Table 3. Both 4-CP and 2,4-DCP levels were detected in the WWTP effluent, however, the concentrations were below the stipulated guideline of 0.01 mg/L set by the South African Department of Water Affairs and Forestry (DWAF, 1996). The levels obtained ranged from 4.04 × 10ˉ⁶ mg/L - 5.61 × 10ˉ⁵ mg/L for 4-CP, and 8.80 × 10ˉ⁷ - 5.40 × 10ˉ⁶ mg/L for 2,4-DCP.

**TABLE 3:** Levels of 4-CP and 2,4-DCP in potable water and WWTP effluent samples in mg/L (mean ± SD, n = 3).

Sample	4-CP					2,4-DCP				
	Sample 1	Sample 2	Sample 3	Mean	SD	Sample 1	Sample 2	Sample 3	Mean	SD
WWTP influent	1.04 × 10ˉ⁴	1.15 × 10ˉ⁴	6.43 × 10ˉ⁵	9.43 × 10ˉ⁵	2.66 × 10ˉ⁵	9.81 × 10ˉ⁵	1.72 × 10ˉ⁴	6.39 × 10ˉ⁵	1.11 × 10ˉ⁵	5.51 × 10ˉ⁵
WWTP effluent	5.61 × 10ˉ⁵	1.27 × 10ˉ⁵	4.04 × 10ˉ⁶	2.43 × 10ˉ⁵	2.79 × 10ˉ⁵	ND	ND	5.40 × 10ˉ⁶	1.80 × 10ˉ⁶	3.12 × 10ˉ⁶
Bottled water brand A	ND	9.32 × 10ˉ⁷	5.81 × 10ˉ⁶	2.25 × 10ˉ⁶	3.12 × 10ˉ⁶	ND	3.68 × 10ˉ⁶	1.31 × 10ˉ⁵	5.60 × 10ˉ⁶	6.77 × 10ˉ⁶
Bottled water brand B	ND	6.95 × 10ˉ⁶	3.42 × 10ˉ⁶	3.46 × 10ˉ⁶	3.48 × 10ˉ⁶	5.56 × 10ˉ⁶	1.37 × 10ˉ⁵	3.68 × 10ˉ⁶	7.66 × 10ˉ⁶	5.35 × 10ˉ⁶
Bottled water brand C	ND	ND	9.78 × 10ˉ⁶	3.26 × 10ˉ⁶	5.64 × 10ˉ⁶	8.80 × 10ˉ⁷	ND	6.85 × 10ˉ⁶	2.58 × 10ˉ⁶	3.73 × 10ˉ⁶
Bottled water brand D	1.97 × 10ˉ⁶	8.90 × 10ˉ⁷	6.74 × 10ˉ⁶	3.20 × 10ˉ⁶	3.11 × 10ˉ⁶	1.47 × 10ˉ⁵	8.37 × 10ˉ⁶	6.28 × 10ˉ⁶	9.77 × 10ˉ⁶	4.36 × 10ˉ⁶
Tap water	9.96 × 10ˉ⁶	1.90 × 10ˉ⁵	ND	9.65 × 10ˉ⁶	9.50 × 10ˉ⁶	6.23 × 10ˉ⁶	1.90 × 10ˉ⁵	5.97 × 10ˉ⁶	9.27 × 10ˉ⁶	5.49 × 10ˉ⁶

ND, not detected.

### 3.2 Carcinogenic risk assessment

The carcinogenic risk assessment result for the water samples is presented in Table 4. The risk assessment table shows the calculated values for ADD, HQ, RfD, SF, and CR. The ADD, representing the quantity of a substance consumed per day during the duration of exposure is a crucial criterion for health risk assessment. The cancer risk assessment was conducted using an assumed exposure period of 10 years, a life expectancy of 70 years, and a body weight of 70 kg. The mean levels of 4-CP and 2,4-DCP obtained from the tap water, bottled water, and effluent samples were used for the cancer risk estimation. Values obtained from the computation of the HQ are used to interpret the cancer risk assessment. A HQ > 1 indicates a carcinogenic adverse effect, whereas HQ < 1 connotes non-carcinogenic adverse effects. The HQ value for all samples was <1; consequently, all the samples are categorized as a non-carcinogenic risk for lifetime exposure.

**TABLE 4:** Cancer risk assessment using mean concentrations of 4-CP and 2,4-DCP of samples.

Sample	ADD	RfD	HQ	SF	CR	Comment
**4-CP**						
WWTP influent	6.15 × 10^−7^	3.00 × 10^−1^	2.05 × 10^−6^	1.10 × 10^−2^	6.76 × 10^−9^	Non-carcinogenic adverse effect
WWTP effluent	1.58 × 10^−7^	3.00 × 10^−1^	5.28 × 10^−7^	1.10 × 10^−2^	1.74 × 10^−9^	Non-carcinogenic adverse effect
BW A	2.35 × 10^−5^	3.00 × 10^−1^	7.82 × 10^−5^	1.10 × 10^−2^	2.58 × 10^−7^	Non-carcinogenic adverse effect
BW B	3.61 × 10^−5^	3.00 × 10^−1^	1.20 × 10^−4^	1.10 × 10^−2^	3.97 × 10^−7^	Non-carcinogenic adverse effect
BW C	3.40 × 10^−5^	3.00 × 10^−1^	1.13 × 10^−4^	1.10 × 10^−2^	3.74 × 10^−7^	Non-carcinogenic adverse effect
BW D	3.34 × 10^−5^	3.00 × 10^−1^	1.11 × 10^−4^	1.10 × 10^−2^	3.67 × 10^−7^	Non-carcinogenic adverse effect
Tap water	1.01 × 10^−4^	3.00 × 10^−1^	3.35 × 10^−4^	1.10 × 10^−2^	1.11 × 10^−6^	Non-carcinogenic adverse effect
**2,4-DCP**						
WWTP influent	7.24 × 10^−8^	3.00 × 10^−1^	2.41 × 10^−7^	1.10 × 10^−2^	7.96 × 10^−10^	Non-carcinogenic adverse effect
WWTP effluent	1.17 × 10^−8^	3.00 × 10^−1^	3.91 × 10^−8^	1.10 × 10^−2^	1.29 × 10^−10^	Non-carcinogenic adverse effect
BW A	5.84 × 10^−5^	3.00 × 10^−1^	1.95 × 10^−4^	1.10 × 10^−2^	6.42 × 10^−7^	Non-carcinogenic adverse effect
BW B	7.9 × 10^−5^	3.00 × 10^−1^	2.66 × 10^−4^	1.10 × 10^−2^	8.79 × 10^−7^	Non-carcinogenic adverse effect
BW C	2.69 × 10^−5^	3.00 × 10^−1^	8.97 × 10^−5^	1.10 × 10^−2^	2.96 × 10^−7^	Non-carcinogenic adverse effect
BW D	1.02 × 10^4^	3.00 × 10^−1^	3.40 × 10^−4^	1.10 × 10^−2^	1.12 × 10^−6^	Non-carcinogenic adverse effect
Tap water	9,67 × 10^−5^	3.00 × 10^−1^	3.22 × 10^−4^	1.10 × 10^−2^	1.06 × 10^−6^	Non-carcinogenic adverse effect

HQ > 1 indicates a carcinogenic adverse effect; HQ < 1 connotes non-carcinogenic adverse effect; BW: bottled water; ADD: average daily dose; RfD: reference dose; HQ; hazard quotient; SF: Slope Factor and CR: cancer risk. Bold values indicate the sectioning of the tables.

### 3.3 Mutagenicity test

The Ames test is a biological test that tests the mutagenic ability of chemical compounds (Zeiger, 2019). It makes use of bacteria to test the potential of chemicals that could have the potential to cause mutations in the DNA of the test organism. Some mutagens may undergo metabolic conversion to a reactive metabolite with the ability to interact with DNA; hence, mutagenicity assay for compounds with or without metabolism is essential for a robust assessment as mammals exhibit extensive *in vivo* metabolic capabilities (EBPI, 2019). The direct or direct mutagens can be detected if S9 is added to the assay mixture, which forms the basis for the addition of the S9 mix. The wells that changed to yellow coloration were considered positive, whereas those that retained the purple color were termed negative. The results were taken at 24 h intervals as presented in Tables 5 and Table 6.

**TABLE 5:** Test scores of samples’ mutagenicity using the T98 strain.

#	Plate	Concentration	Bacteria	Day 4	Day 5	Day 6
1	Blank –ws9 (tap water)	100%	-	0	0	0
2	Background wS9	-	+	0	0	0
3	Positive control	-	+	95	96	96
4	WWTP effluent I	100%	+	91	91	91
5	WWTP effluent-I ws9	100%	+	95	95	95
6	WWTP effluent-II	19%	+	96	96	96
7	Tap water I	100%	+	95	95	95
8	Tap water-I ws9	100%	+	95	95	95
9	Tap water –II	19%	+	95	95	95
10	Bottled water ‘A’-I	100%	+	96	96	96
11	Bottled water ‘A’- I wS9-	100%	+	96	96	96
12	Bottled water ‘A’-II	19%	+	96	96	96
13	Bottled water ‘B’-I	100%	+	96	96	96
14	Bottled water ‘B’-I wS9	100%	+	96	96	96
15	Bottled water ‘B’-II	19%	+	96	96	96

**TABLE 6:** Test scores of samples’ mutagenicity using the T100 strain.

#	Plate	Concentration	Bacteria	Day 4	Day 5	Day 6
1	Blank –wS9 (tap water)	100%	-	0	0	0
2	Background wS9	-	+	19	21	23
3	Positive Control	-		54	64	68
4	WWTP effluent I	100%	+	30	35	38
5	WWTP effluent-I ws9	100%	+	26	29	30
6	WWTP effluent-II	19%	+	25	32	35
7	Tap water I	100%	+	29	33	39
8	Tap water-I ws9	100%	+	19	21	24
9	Tap water –II	19%	+	21	33	35
10	Bottled water ‘A’-I	100%	+	16	19	24
11	Bottled water ‘A’- I wS9-	100%	+	30	37	39
12	Bottled water ‘A’-II	19%	+	13	20	23
13	Bottled water ‘B’-I	100%	+	25	30	32
14	Bottled water ‘B’-I wS9	100%	+	19	23	26
15	Bottled water ‘B’-II	19%	+	15	17	23

The T98 bacterial strain results are considered valid when three criteria are met. These criteria include ensuring that the blank wells are sterile; the mean score for the background control (negative control) is ≥0 and ≤30 revertant wells per 96-well section on day 6; and the mean score for the standard mutagen (positive control) is ≥50 revertant wells per 95-well section on day 6.

## 4 Discussion

### 4.1 Phenol occurrence in WWTP effluent and potable water samples

Processing chemicals, raw materials, or intermediate products in the agrochemical industry and wood preservation are sources of phenolic compounds in the environment (Yahaya et al., 2019; Ramos et al., 2021). Chlorophenols are produced in pulp bleaching processes, as metabolites of agricultural pesticides, and as by-products of chlorination during water/wastewater treatment operations (Yahaya et al., 2019). The WWTP is situated in the Boland region of the Western Cape Province in South Africa-a vast area that is known for its agricultural process as numerous commercial farms abound. These farms mostly grow grapevines used to produce different types of wines and their farming operation requires the use of several pesticides. The presence of the low levels of 2,4-DCP may be from the agrochemical usage from these farms as the WWTP solely uses the membrane bioreactor in its treatment process. The membrane reactor system does not require chlorine usage for effluent treatment. Rather, it uses a combination of biological methods for suspended growth (generally activated sludge) and membrane filtration operations; hence, no chlorination by-product is generated. Essentially, low-pressure microfiltration or ultrafiltration is utilized to carry out critical solid-liquid separation functions (AMTA, 2016; Nqombolo et al., 2018). Therefore, the levels of 2,4-DCP in the effluent samples are expected to be low due to this process of treatment used because the 2,4-DCP compound in drinking and wastewater occurs mostly as a by-product of water treated by chlorination (Park and Kim, 2018). For 4-CP, the wine production (wineries) in the surrounding areas of the WWTP could be the major contributor to the trace levels detected in effluent samples (Girish and Murty, 2012). Winery effluent which majorly comes from washing operations during grapes harvesting, pressing, and fermentation contains low levels of phenolic compounds (Cassano et al., 2015).

Four brands of bottled water denoted as brands “A, B, C, and D” were assessed for their phenolic content. The maximum concentrations of 4-CP and 2,4-DCP recorded for all the bottled water samples were 9.78 × 10ˉ⁶ mg/L and 1.47 × 10ˉ⁵ mg/L, respectively. The guideline for phenol in bottled water as stipulated by the United States Food and Drug Administration (US FDA) is 0.001 mg/L (ATSDR, 2008); the levels obtained for both compounds were below the FDA regulatory limits. A previous investigation on the USEPA 11 priority phenols in three brands of bottled water in Cape Town, South Africa recorded an average concentration of 5.13 × 10^−3^ mg/L. In the tap water samples, the concentration of 4-CP ranged from 9.96 × 10^−6^ - 1.90 × 10^−5^ mg/L, while 2,4-DCP was from 5.97 × 10^−6^ - 1.90 × 10^−5^ mg/L. The United States Environmental Protection Agency (USEPA) maximum permissible limit for phenol in potable water is ≤0.3 mg/L, stipulated for the protection of human health from potential adverse effects of phenol exposure via drinking water and/or contaminated animals and plants (Zeatoun et al., 2004). Also, the European Community stipulates a concentration of 0.1 μg/L as the maximum permissible limit for phenol in drinking water (Al-Janabi, 2011). The agencies set benchmarked concentrations for contaminants as maximum permissible limits to prevent adverse human health effects. The measured levels of both phenolics in the tap water were below the set limits which connotes potability. The likely sources of the compounds in the tap water could be from the chlorination process employed for water disinfection. More so, the raw water sources utilized by most water works come from dams which are filled up during the winter season by run-offs which can be contaminated by agricultural chemicals. Aizawa et al. (2015) recorded concentrations in the ranges of 0.01—0.20 mg/L for phenols in tap water; the study did not differentiate between the individual phenolic compounds.

### 4.2 Non-carcinogenic and carcinogenic risk assessment

The phenol concentrations in the water samples utilized for the cancer risk assessment were all below the stipulated guidelines set by national and international bodies. The guideline set for phenols in WWTP effluent by the Department of Water Affairs and Forestry (DWAF) in South Africa is 0.01 mg/L (DWAF, 1996). The US FDA guideline stipulates a maximum permissible limit of 0.001 mg/L in bottled water, with a lifetime exposure of 2 mg/L not expected to cause an adverse health effect (ATSDR, 2008). The European Union limit is 0.5 μg/L for total phenols and 0.1 μg/L for individual compounds (Fattahi et al., 2007; Mahugo Santana et al., 2009). Contaminants with concentrations exceeding the maximum permissible limits stipulated by environmental protection bodies pose adverse health implications. The trace levels of 4-CP (9.32 × 10^−7^—1.15 × 10^−4^ mg/L) and 2,4-DCP (8.80 × 10^−7^—1.72 × 10^−4^ mg/L) recorded in the water samples translate to the non-carcinogenic risk recorded. None of the samples analyzed had a cancer risk (CR) value that exceeded any of the regulatory limits. Typically, HQ values > 1 portend potential harmful ecological effects, whereas values < 1 suggest a low possibility of ecological risk (Megahed et al., 2015). The HQ values recorded in this study were all <1, indicating a low level of potential ecological risk. More so, the recorded ADD values for the phenolics were below 10^–4^, which indicates a low possibility of cancer risk (Yahaya et al., 2019). Cancer risk values < 10^–6^ indicate no likelihood of cancer (Ramos et al., 2021); none of the samples analyzed had a cancer risk value that exceeded the regulatory limit. Therefore, the possibility of the analyzed samples causing cancer is unlikely. Despite the low possibility of cancer risk observed, cognizance of the assessment being done with data for a healthy adult should be noted. This implies that the observed values may pose a cancer risk to children and other adults with compromised or debilitating health conditions.

### 4.3 Mutagenicity assessment

The assay results show that the background control had no (0) positive well, while the bottled water “A” and “B”, tap water, and the WWTP effluent of the undiluted concentrations (100%) recorded 96, 96, 95, and 95 positive revertant colonies on the test plates on day 6, respectively. The positive wells indicate that the levels of 4-CP and 2,4-DCP in the water samples are mutagenic and could act as a carcinogen. Using the scoring tables in a 96-well microplate, there was a clear significance in the Fluctuation Test which indicates the strong mutagenicity of the water samples. The assay results for all the undiluted samples (i.e. 96, 96, 95, and 95) are much higher than the control; hence, there is < 0.001 chance that 0 and 95 or 96 are the same results. The treatment plates produced a significant difference in the reverse mutation rate when compared with the control, indicating that the samples assayed possess mutagenicity on the T98 strain. Statistically, increases in the frequency of revertant colonies compared to the concurrent control with *p* < 0.05 indicate significant variation (Wisher, 2017). Fluctuation test is used to assess the mutagenicity of a particular chemical or treatment over a control; it takes cognizance of the number of positive wells in the treatment to that of the control (Gilbert, 1980).

The mutagenicity results showed that the background control had 23 positive wells at day 6, whereas the bottled water “A” and “B”, tap water, and effluent samples recorded 39, 26, 24, and 30 positive wells, respectively. Comparing the number of positive wells in the samples with that of the backgrounds shows significant variation in the Fluctuation Test - an indication of the samples displaying weak mutagenicity. There is < 0.05 chance that the results of bottled water “B” (26), tap water (24), and effluent (30) are the same result as the background control (23), while the bottled water “A” (39) had a <0.001 chance; suggesting a slight chance of mutagenicity for the T100 strain. Mortelmans and Zeiger (2000) suggested that a substance can be categorized as a mutagen when one or more strains induce a reproducible, dose-related increase in the number of reverting colonies; however, a substance is regarded as a poor mutagen when the number of reverting colonies does not double that of the background number of colonies. Essentially, only a sample with twice the number of reverse mutations compared to the background mutation rate is considered to be mutagenic. A positive bacterial reverse mutation assay result suggests that a substance can potentially cause point mutations in the genome of either *S. typhimurium* by base substitution or frameshift (OECD, 1997). Conversely, negative results suggest that the test substance under the defined conditions is not mutagenic in the tested organisms.

## 5 Conclusion

The quantification and human health risk assessment of 4-CP and 2,4-DCP compounds in potable and treated waters in the Boland region of the Western Cape of South Africa was carried out in this study. The levels of 4-CP and 2,4-DCP measured in the samples were below the stipulated limits recommended by national and international bodies. The potential risks of the phenolic levels to humans were investigated using the cancer risk and mutagenicity assays. The assessed HQ for the phenolic concentrations indicates a low level of potential ecological risk. There was no cancer risk associated with the samples but signs of non-carcinogenic adverse effects were found. The mutagenicity assay of 4-CP and 2,4-DCP using the T98 strains recorded strong mutagenicity as there was a significant variation (*p* < 0.05) in the frequency of revertant colonies compared to the concurrent control. The T98 results indicate the potential ability of phenolic concentrations in the water samples to cause mutation toward the insertion or deletion of a nucleotide. However, the T100 bacterial strain showed very slight mutagenicity potential, with no real threat or possibility of causing mutation involving the replacement or substitution of a nucleotide base with another in the DNA or RNA.

This investigation is an introductory study into the health risk assessment of selected phenolic compounds in potable water and treated effluent samples. The study has provided some insight into the possible human health risks associated with the occurrence of the 4-CP and 2,4-DCP water samples. The health risk studies were carried out at population and organism levels; hence, cellular and molecular-level studies may provide greater clarity on the associated health risks of the phenolic compounds at the observed concentrations.

## Data Availability

The original contributions presented in the study are included in the article/Supplementary Material, further inquiries can be directed to the corresponding author.
