# Supplementary material for: Phenolic compounds occurrence and human health risk assessment in potable and treated waters in Western Cape, South Africa
Source: Front Toxicol. 2024 Jan 4;5:1269601. doi: 10.3389/ftox.2023.1269601 (PMC10794607; doi:10.3389/ftox.2023.1269601)
Supplement: Supplementary file 1 [file Table1.docx]

Supplementary Material

**Phenolic compounds occurrence and human health risk assessment in potable and treated waters in Western Cape, South Africa**

**Nkosiyenzile Londiwe Mhlongo^1^, Michael Ovbare Akharame^2^*, Omoniyi Pereao^1^, Izanne Susan Human^1^, Beatrice Olutoyin Opeolu^3^**

**^1^**Department of Environmental and Occupational Studies, Cape Peninsula University of Technology, Cape Town 8000, South Africa

**^2^**Department of Environmental Management and Toxicology, University of Benin, Benin-City 3002, Nigeria

^3^Environmental Chemistry and Toxicology Research Group, Cape Peninsula University of Technology, Cape Town 8000, South Africa

*** Correspondence:**Corresponding Author: Michael Ovbare Akharame
 email: michael.akharame@uniben.edu

**SI Table 1: Chromatographic parameters for quantification of phenols**

| Chromatograph | Waters | | |  |
| --- | --- | --- | --- | --- |
| Detector | UV | | |  |
| Column | Ace 5 C18 column, 3.9mm 5μ | | | |
| Injection volume | 20 μL | | |  |
| Mobile phase | A: water | | |  |
|  | B: Acetonitrile | | |  |
| Flow-rate | 1 mL/min | | |  |
| Gradient elution | Time (mins) | %A | %B |  |
|  | 0 | 85 | 15 |  |
|  | 25 | 0 | 100 |  |
|  | 28 | 0 | 100 |  |
|  | 30 | 85 | 15 |  |
| Temperature | 25 °C | | |  |
| Data Collection | Breeze software version 2 | | |  |

**SI Table 2:** Exposure values used in dose calculation

| **Exposure parameter** | **Value** |
| --- | --- |
| Exposure duration (ED)- years | 10 |
| Body weight (bw) kg | 70 |
| Lifetime (LT) years | 70 |
| Exposure frequency (EF) days/years | 45 |
| Exposure time (ET) hour/event | 1 |
| Events/day EV | 1 |
| Water intake rate L/hr | 0.071 |

**SI Fig 1:** Chromatogram of 4-CP and 2,4-DCP

**SI Fig. 2a**: Calibration curve for 4-CP

**SI Fig. 2b**: Calibration curve for 2,4-DCP

**SI Table 3:** Calibration data for 4-CP and 2,4-DCP

| **4-CP** | | | **2,4-DCP** | | |
| --- | --- | --- | --- | --- | --- |
| **Concentration (M)** | **Peak Area** | **Retention Time** | **Concentration (M)** | **Peak Area** | **Retention time** |
| 0.00006 | 113374 | 11.715 | 0.00006 | 123054 | 14.059 |
| 0.0003 | 557496 | 11.767 | 0.0003 | 614290 | 14.122 |
| 0.0009 | 1648622 | 11.761 | 0.0009 | 1805903 | 14.119 |
| 0.0012 | 2193595 | 11.762 | 0.0012 | 2421714 | 14.118 |
| 0.0015 | 2759050 | 11.723 | 0.0015 | 3058599 | 14.073 |
| 0.0018 | 3276298 | 11.739 | 0.0018 | 3610620 | 14.070 |
| 0.0021 | 3815336 | 11.708 | 0.0021 | 4224544 | 14.070 |
| 0.003 | 5420296 | 11.719 | 0.003 | 5969507 | 14.081 |
